# Grouper TRIM23 exerts antiviral activity against iridovirus and nodavirus

**DOI:** 10.3389/fimmu.2022.985291

**Published:** 2022-09-20

**Authors:** Linyong Zhi, Wenji Wang, Jiaying Zheng, Shanxing Liu, Sheng Zhou, Qiwei Qin, Youhua Huang, Xiaohong Huang

**Affiliations:** ^1^ Guangdong Laboratory for Lingnan Modern Agriculture, College of Marine Sciences, South China Agricultural University, Guangzhou, China; ^2^ University Joint Laboratory of Guangdong Province, Hong Kong and Macao Region on Marine Bioresource Conservation and Exploitation, Guangzhou, China; ^3^ Southern Marine Science and Engineering Guangdong Laboratory, Zhuhai, China

**Keywords:** grouper, EcTRIM23, fish virus, TBK1, TRAFs, interferon

## Abstract

TRIM (tripartite motif) proteins have been demonstrated to exert critical roles in host defense against different microbial pathogens. Among them, TRIM23 acts as an important regulatory factor in antiviral immune and inflammatory responses, but the roles of fish TRIM23 against virus infection still remain largely unknown. Here, we investigated the characteristics of TRIM23 homolog from orange spotted grouper (*Epinephelus coioides*) (EcTRIM23). EcTRIM23 encoded a 580 amino acid peptide, which shared 93.1%, 89.73% and 86.36% identity with golden perch (*Perca flavescens*), zebrafish (*Danio rerio*) and human (*Homo sapiens*), respectively. The transcription levels of EcTRIM23 were significantly up-regulated in response to Singapore grouper iridovirus (SGIV) and red-spotted grouper nervous necrosis virus (RGNNV) infection. EcTRIM23 overexpression *in vitro* significantly inhibited RGNNV and SGIV replication, evidenced by the delayed cytopathic effect (CPE) progression and the decreased expression of viral core genes. EcTRIM23 significantly increased the expression levels of interferon (IFN) related signaling molecules and pro-inflammatory cytokines, as well as the promoter activities of IFN and NF-κB, suggesting that EcTRIM23 exerted antiviral function by positively regulating host IFN response. Exogenous EcTRIM23 exhibited either diffuse or aggregated localization in grouper cells. After co-transfection, TANK binding kinase 1 (TBK1), TNF receptor associated factor (TRAF) 3 and TRAF4, TRAF5 and TRAF6 were found to interact with EcTRIM23 in grouper cells. Moreover, these proteins could be recruited and co-localized with EcTRIM23 *in vitro*. Together, our results demonstrated that fish TRIM23 exerted antiviral activity against fish viruses by interacting with multiple host proteins to regulate immune responses.

## Introduction

Tripartite motif-containing (TRIM) proteins are a versatile family of E3 ligases which are involved in a multitude of cellular processes, including cell proliferation, differentiation, growth, apoptosis and cancer, signal transduction and innate immune responses (1–4). Notably, recent studies have demonstrated that many TRIM proteins play important roles in the host defense against viral infection, and most of them have emerged as key components of the innate immune system (1, 3). For instance, TRIM25 E3 ubiquitin ligase induced the Lys 63-linked ubiquitination of retinoic acid-inducible gene I (RIG-I) to elicit host antiviral innate immunity (5), and TRIM21 overexpression resulted in the secretion of tumor necrosis factor alpha (TNF-α) and interleukin-6 (IL-6) by activating the proinflammatory response (6). TRIM65-catalized ubiquitination was essential for melanoma differentiation associated gene 5 (MDA5)-mediated antiviral innate immunity (7). In addition, TRIM5α also interacted with the intact HIV-1 capsid lattice and formed a complementary lattice that induced premature virion disassembly and blocked viral infection (8). Interestingly, the great diversity and considerable variation in the number of TRIM genes were detected in the genomes of teleost fish (9, 10). Moreover, zebrafish finTRIM83 induced IFN and IFN-stimulated gene expression and afforded protection against viral hemorrhagic septicemia virus (VHSV) and infectious hematopoietic necrosis virus (IHNV) infection (11, 12).

TRIM23, also named ADP ribosylation factor domain protein 1 (ARD1), has been reported to exhibit two enzymatic functions: E3 ubiquitin ligase activity in the RING domain and GTPase activity in the C-terminal ARF domain (13, 14). Subcellular localization showed that TRIM23 was associated with the Golgi complex and lysosomal structures (15). TRIM23 interacted with TBK1, which was well known to regulate the IFN response and recently had been related to autophagy (16). In addition, ubiquitin conjugation mediated by TRIM23 to the NF-κB essential modulator was important to antiviral innate and inflammatory responses mediated by toll-like receptors 3 (TLR3) and RIG-I/MDA5 (17, 18). In addition to interacting with host proteins, TRIM23 also affected the host interferon immune response by interacting with viral proteins. Herpes simplex virus 1 (HSV-1) Us11 protein targeted TRIM23 and disrupted the TRIM23-TBK1 complex, which impaired autophagy and autophagy-mediated virus restriction (19). Although great efforts have been made on the roles of mammalian TRIM23 in response to virus infection, few reports focused on the function of TRIM23 from lower vertebrates, especially from fish (20).

Groupers (*Epinephelus* spp.) are widely cultured in China and south-east Asian countries. However, the emergence of iridoviral and nodaviral diseases caused heavy economic losses in grouper aquaculture in recent years (21, 22). Singapore grouper iridovirus (SGIV), a large DNA virus which belongs to family *Iridoviridae*, induced the mortality rates from 30% (adult fish) to 100% (fry). Red-spotted grouper nervous necrosis virus (RGNNV), a non-enveloped RNA virus which belongs to family *Nodaviridae* induced up to 100% mortality rates at the larval and juvenile stages in grouper aquaculture (23). To date, multiple host immune regulatory molecules involved in TLR signaling pathway have been characterized in grouper defense against iridovirus and nodavirus pathogens, including stimulator of interferon genes (STING) (24), TBK1 (25), mitochondrial antiviral signaling protein (MAVS) (26), MDA5 (27), laboratory of genetics and physiology 2 (LGP2) (28), and tumor necrosis factor receptor-associated factors (TRAFs) (29, 30). In addition, several TRIMs were cloned from grouper and their roles in fish viruses replication were characterized. For example, grouper TRIM13, TRIM62 and TRIM35 were found to negatively regulate the antiviral immune response against nodavirus (31–33), whereas grouper TRIM32 and TRIM82 acted as an antiviral factor against both iridovirus and nodavirus infection, respectively (34, 35). Recently a study showed that TRIM23 from grass carp not only colocalized and interacted with TRAF6 and myeloid differentiation factor 88 (MyD88), but also induced autophagy (20). Whether grouper TRIM23 was involved in different fish viruses infection, and the potential mechanism still remained uncertain.

In the present study, we cloned and characterized a novel TRIM23 gene from orange spotted grouper (*Epinephelus coioides*) (EcTRIM23). The expression profiles of EcTRIM23 upon grouper virus infection were examined, and its roles in virus replication was investigated *in vitro*. Furthermore, the regulatory effects of EcTRIM23 on host immune genes were evaluated and its interacted proteins were identified. Our results will provide new insights into understanding the mechanism of fish TRIM23 against viral infection.

## Materials and methods

### Cells and viruses

Grouper spleen (GS) cells were maintained in Leibovitz’s L15 medium supplemented with 10% fetal bovine serum (FBS, Gibco) at 28°C (36). The viruses including SGIV and RGNNV were infected with GS cells, collected and lysed by three freeze-thaw cycles. The virus stocks were titered by the 50% tissue culture infective dose (TCID50) assay as described previously (36).

### EcTRIM23 cloning, sequence analysis and plasmid construction

Based on the EST sequences from the transcriptome data (36, 37), the ORF of EcTRIM23 was cloned by PCR amplification using primers EcTRIM23-ORF-F/EcTRIM23-ORF-R, and then verified using DNA sequencing. The conserved functional domains were predicted using SMART program. Multiple sequence alignment was performed using ClustalX1.83 software, and edited with GENEDOC program. A Neighbor-joining phylogenetic tree was constructed using Mega 6.0 software.

To clarify the potential function of EcTRIM23 *in vitro*, EcTRIM23 was cloned into pEGFP-C1 vector using the specific primers EcTRIM23-C1-HindIII-F/EcTRIM23-C1-BamHI-R. The recombinant plasmid (pEGFP-EcTRIM23) was subsequently confirmed by DNA sequencing.

### Expression profiles of EcTRIM23 in response to viral infection

To determine the expression patterns of EcTRIM23 against fish virus infection, GS cells were infected with SGIV or RGNNV at a multiplicity of infection (MOI) of 2. Then cells were collected at 6 h, 12 h, 24 h, 48 h post-infection (p.i.), and subjected to further quantitative PCR (qPCR) analysis.

### Cell transfection and immune fluorescence assay

To detect the localization of EcTRIM23 *in vitro*, GS cells were seeded into 24-well plates overnight, and then transfected with pEGFP-C1 or pEGFP-EcTRIM23 using the transfection reagent Lipofectamine 2000 (Invitrogen) according to the manufacturer’s instructions (31). At 48 h post-transfection (p.t.), cells were fixed with 4% paraformaldehyde (PFA) and stained with 4, 6-diamidino-2-phenylindole (DAPI) for 5 min. The cells were observed under fluorescence microscopy (Zeiss, Germany).

To determine the levels of co-localization between EcTRIM23 and its interacted partners, GS cells were co-transfected pEGFP-EcTRIM23 with HA-EcTBK1, HA-EcTRAF3, HA-EcTRAF4, HA-EcTRAF5 or HA-EcTRAF6, respectively. The intracellular localization of HA-tagged proteins were processed using immunofluorescence assay (IFA) as described previously (38). At 48 h p.t., cells were fixed and incubated with anti-HA (1:200), followed by goat anti-mouse IgG Fab2 Alexa Fluor 555 (Invitrogen, USA; 1:200). Finally, cells were stained with DAPI and observed under a fluorescence microscope (Zeiss, Germany).

### RNA extraction and qPCR analysis

To determine the effects of EcTRIM23 on viral or host gene transcriptions, the transfected or infected cells were harvested at indicated time points for RNA isolation. The total RNA of cells was extracted using an SV Total RNA Isolation System (Promega) and reverse-transcribed with a ReverTra Ace qPCR RT kit (Toyobo) as described previously (31). The transcription levels of viral or host genes were determined by qPCR using the SYBR Green real-time PCR Kit (Toyobo) according to the manufacturers’ instructions. The viral genes included RGNNV coat protein (CP), RNA-dependent RNA polymerase (RdRp) and SGIV major capsid protein (MCP), and viral protein VP19. The host immune genes included interferon regulator factor (IRF) 3, IRF7, interferon stimulated gene (ISG) 15, ISG56, interferon-induced 35 (IFP35), and myxovirus resistance gene (MXI), interleukin (IL)-1β, IL-8, and tumor necrosis factor α (TNFα). The primers used in this study were listed in **Table 1**. Each qPCR analysis was performed at least in triplicate using the following cycling conditions: 94°C for 5 min, followed by 45 cycles at 94°C for 5 s, 60°C for 10 s, and 72°C for 15 s. The levels of target gene expression were normalized to that of β-actin and calculated using the 2^−ΔΔCT^ method. The data were represented as the mean ± standard deviation (SD).

**Table 1:** Primers used in this study.

Primer names	Sequence5’-3’
EcTRIM23-ORF-F	ATGGCCGCTGCTGCAGCAG
EcTRIM23-ORF-R	GGCCACGTCCAGGACGC
EcTRIM23-C1-HindIII-F	*AAGCTT*CGATGGCCGCTGCTGCAGCAG
EcTRIM23-C1-BamHI-R	*GGATTC*GGCCACGTCCAGGACGC
EcTRIM23-RT-F	GCGGTGGTGTTCGTGATT
EcTRIM23-RT-R	GCAAAGATGAGCAGCAAGG
Actin- RT-F	TACGAGCTGCCTGACGGACA
Actin- RT-R	GGCTGTGATCTCCTTCTGCA
RGNNV CP-RT-F	CAACTGACAACGATCACACCTTC
RGNNV CP-RT-R	CAATCGAACACTCCAGCGACA
RGNNV RdRp-RT-F	GTGTCCGGAGAGGTTAAGGATG
RGNNV RdRp-RT-R	CTTGAATTGATCAACGGTGAACA
SGIV MCP- RT-F	GCACGCTTCTCTCACCTTCA
SGIV MCP- RT-R	AACGGCAACGGGAGCACTA
SGIV VP19-RT-F	TCCAAGGGAGAAACTGTAAG
SGIV VP19-RT-R	GGGGTAAGCGTGAAGAC
EcTNFα-RT-F	GTGTCCTGCTGTTTGCTTGGTA
EcTNFα-RT-R	CAGTGTCCGACTTGATTAGTGCTT
EcIL-1β-RT-PF	AACCTCATCATCGCCACACA
EcIL-1β-RT-PR	AGTTGCCTCACAACCGAACAC
EcIL-8-RT-PF	GCCGTCAGTGAAGGGAGTCTAG
EcIL-8-RT-PR	ATCGCAGTGGGAGTTTGCA
EcMXI-RT-F	CGAAAGTACCGTGGACGAGAA
EcMXI-RT-R	TGTTTGATCTGCTCCTTGACCAT
EcISG15-RT-F	CCTATGACATCAAAGCTGACGAGAC
EcISG15-RT-R	GTGCTGTTGGCAGTGACGTTGTAGT
EcIRF3-RT-F	GACAACAAGAACGACCCTGCTAA
EcIRF3-RT-R	GGGAGTCCGCTTGAAGATAGACA
EcIRF7-RT-F	CAACACCGGATACAACCAAG
EcIRF7-RT-R	GTTCTCAACTGCTACATAGGG
EcIFP35-RT-F	TTCAGATGAGGAGTTCTCTCTTGTG
EcIFP35-RT-R	TCATATCGGTGCTCGTCTACTTTCA
EcISG56-RT-F	CAGGCATGGTGGAGTGGAAC
EcISG56-RT-R	CTCAAGGTAGTGAACAGCGAGGTA

### Dual-luciferase reporter assay

To detect the interferon promoter activity induced by EcTRIM23, the reporter plasmids (IFN-Luc, ISRE-Luc or NF-κB-Luc) or Renilla luciferase (internal control) were co-transfected with pEGFP-EcTRIM23 or pEGFP-C1 in grouper cells, respectively. At 48 h p.t., cells were lysed, and the luciferase assay was determined using the Dual-Luciferase Reporter Assay system (Promega) as described previously (31).

### Co-Immunoprecipitation (Co-IP) assay and immunoblotting analysis

For the Co-IP assay, GS cells were seeded into 10-cm dishes overnight and co-transfected with target gene plasmids, including 3HA-EcTBK1, 3HA-EcTRAF3, 3HA-EcTRAF4, 3HA-EcTRAF5 or 3HA-EcTRAF6, and pEGFP-EcTRIM23 or pEGFP-C1. At 48 h p.t., cells were washed twice with ice-cold PBS, and then lysed by IP lysis buffer. After centrifugation at 12,000 g for 3 min at 4°C, the supernatants were collected for immunoprecipitation using the Dynabeads™ Protein G Immunoprecipitation Kit (Thermofisher, USA) according to the instructions. In brief, the Dynabeads™ Protein G were incubated with anti-GFP (1:200; Abcam, USA) for 10 min at room temperature and incubated with the supernatants containing the antigen (Ag) for 90 min. After washing with washing buffer, the Dynabeads-Ab-Ag complex was gently resuspended in 20 µl elution buffer for immunoblotting assay with indicated Abs (37).

The complex mentioned above was separated by 10% sodium dodecyl sulfate polyacrylamide gel electrophoresis (SDS-PAGE), and then transferred to 0.22-μm polyvinylidene difluoride (PVDF) membranes (Millipore, USA). After blocking with 5% skim milk, the membranes were incubated with the anti-GFP (1:1,000; Abcam, USA), anti-HA (1:1,000; Sigma, USA) or anti-β-tubulin (1:2,000; Abcam, USA) for 2 h. Subsequently, the membranes were incubated with horseradish peroxidase (HRP)-conjugated sheep anti-rabbit IgG or sheep anti-mouse IgG (1:3,000; Abcam, USA) for further 2 h. After washing with PBST, the specific binds were visualized using Pierce™ ECL Western Blotting Substrate (Thermofisher, USA). The results were representative of three independent experiments.

### Statistical analysis

The statistical analysis was performed using SPSS version 20. Statistical significance was determined using a student’s t-test and established at p < 0.05(*).

## Results

### Sequence characteristics of EcTRIM23

**Figure 1:**
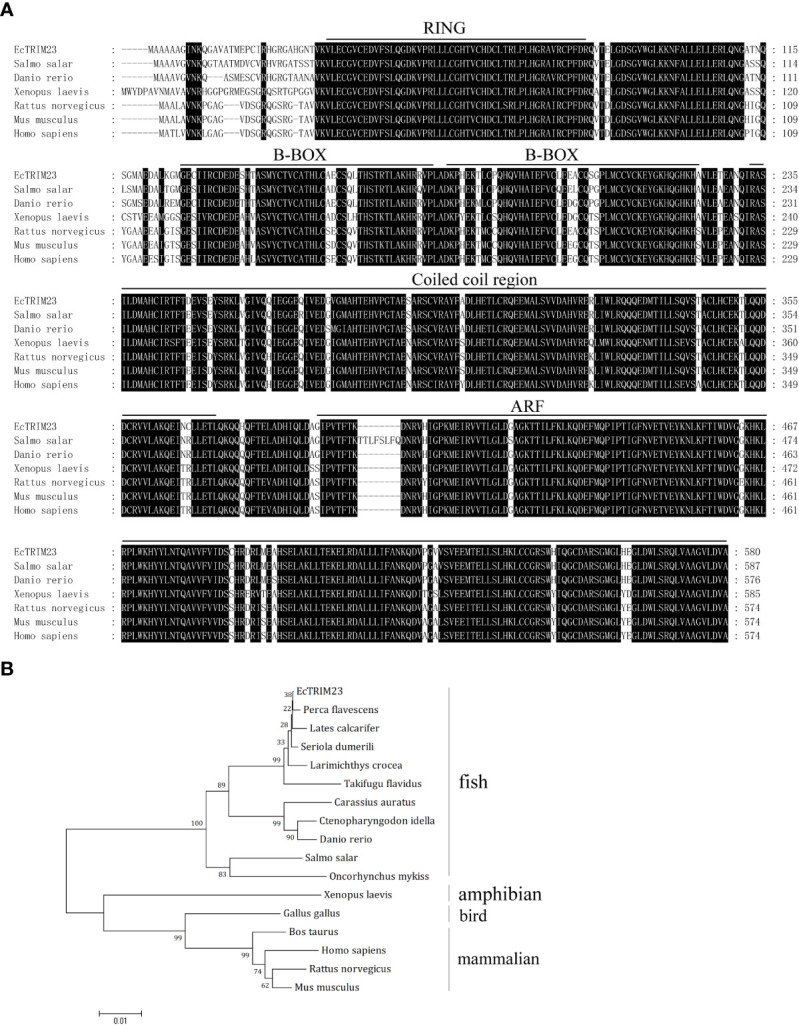
Sequence characteristics of EcTRIM23. **(A)** Amino acid alignment of EcTRIM23 and other TRIM23 homologs from different species. The conserved domains, including RING domain, B-BOX domain, coiled-coil domain and ARF domain are underlined. **(B)** Phylogenetic analysis of EcTRIM23. A phylogenetic tree was constructed using MEGA 4.0 with the neighbor-joining (NJ) method. The bootstrap values are indicated at the branch points. The sequences of TRIM23 genes used in this study were obtained from GenBank, and their accession numbers are listed as follows: *Perca flavescens*, XP_028457727; *Lates calcarifer*, XP_018517789; *Seriola dumerili*, XP_022595517; *Larimichthys crocea*, XP_019134261; *Takifugu flavidus*, TWW66274; *Carassius auratus*, XP_026129098; *Ctenopharyngodon idella*, QBQ04133; *Danio rerio*, XP_005155659; *Salmo salar*, XP_013982389; *Oncorhynchus mykiss*, XP_021458645; *Xenopus laevis*, XP_018121234; *Gallus gallus*, XP_424752; *Bos taurus*, XP_010815025; *Homo sapiens*, NP_001647; *Rattus norvegicus*, NP_001094107; *Mus musculus*, AAH56390.

After PCR amplification and DNA sequencing, we confirmed that EcTRIM23 encoded a 580 amino acid peptide which shared 93.1%, 89.73% and 86.36% identity with golden perch (*Perca flavescens*), zebrafish (*Danio rerio*) and humans (*Homo sapiens*), respectively. Amino acid alignment indicated that EcTRIM23 contained several conserved domains, including a RING domain, B-BOX domain, coiled-coil domain, and an ADP-ribosylation factor (ARF) domain (**Figure 1A**). Phylogenetic analysis indicated that EcTRIM23 showed the closest phylogenetic relationship to golden perch, followed by other fishes, amphibians and mammals (**Figure 1B**).

### Expression patterns of EcTRIM23 in response to fish viruses *in vitro*

**Figure 2:**
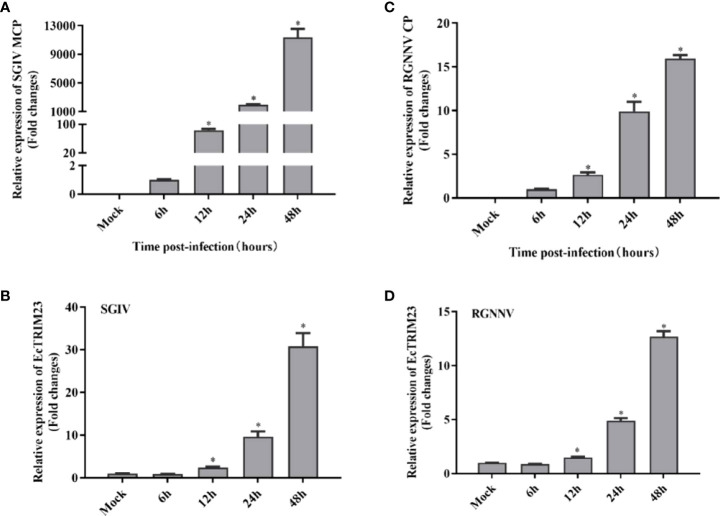
Expression profiles of EcTRIM23 during virus infection. In SGIV or RGNNV infected cells, SGIV MCP **(A)**, RGNNV CP **(B)** and EcTRIM23 **(C, D)** were detected by qPCR. GS cells were infected with SGIV or RGNNV, and then collected at the indicated time points for qPCR examination. *p < 0.05.

To detect the changes in mRNA expression of EcTRIM23 in response to viral infection *in vitro*, GS cells were infected with SGIV or RGNNV, respectively, and the mock- and infected-cells were harvested at the indicated time points for qPCR analysis. As shown in **Figure 2**, the transcription levels of SGIV MCP and RGNNV CP increased gradually with time after infection (**Figures 2A, C**), suggested that SGIV and RGNNV replicated well in GS cells. During SGIV infection, the transcription of EcTRIM23 significantly increased from 12 h p.i., and reached a peak of 30.77-fold at 48 h p.i. compared to mock-infected cells (**Figure 2B**). Similarly, in RGNNV-infected cells, the transcription of EcTRIM23 also gradually increased up to the peak of 12.70-fold at 48 h p.i. compared to that in mock-infected cells (**Figure 2D**). Thus, we speculated that either SGIV or RGNNV infection induced significant increase of EcTRIM23 transcription *in vitro*.

### EcTRIM23 acted as an antiviral factor during SGIV and RGNNV infection

**Figure 3:**
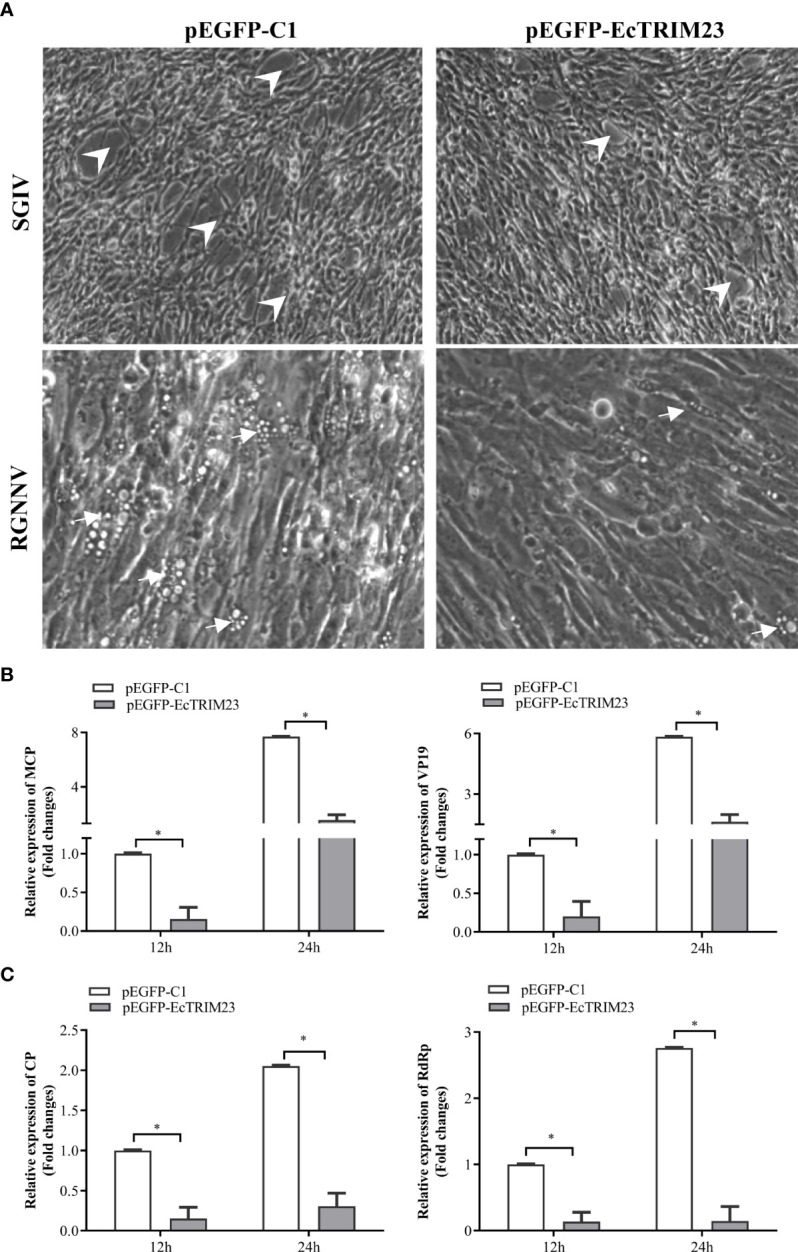
The effects of EcTRIM23 on SGIV and RGNNV replication. **(A)** EcTRIM23 overexpression weakened the severity of virus-induced CPE in GS cells. The white arrows indicated the vacuoles induced by RGNNV infection and the arrowheads showed the rounding and aggregated cells induced by SGIV infection. **(B)** The transcription of SGIV MCP and VP19 in infected EcTRIM23-overexpressing cells. **(C)** The transcription of RGNNV CP and RdRp in infected EcTRIM23-overexpressing cells. The transfected cells were infected with SGIV or RGNNV, and harvested at 12 h.p.i. and 24 h.p.i. to determine the mRNA expression levels of viral genes by qPCR. *p < 0.05.

To clarify the role of EcTRIM23 during virus replication *in vitro*, we evaluated its effects on CPE progression and viral gene transcriptions in EcTRIM23-overexpressing cells upon virus infection. As shown in **Figure 3A**, the severity of SGIV-induced CPE was obviously weakened in EcTRIM23-overexpressing cells compared with the empty vector transfected cells. Consistently, the transcription levels of viral genes, including SGIV MCP and VP19, were significantly decreased in SGIV-infected EcTRIM23-overexpressing cells compared to those in the empty vector transfected cells (**Figure 3B**). Similarly, EcTRIM23 overexpression obviously decreased the number of vacuoles induced by RGNNV, and significantly reduced the expression levels of RGNNV CP and RdRp compared to the empty vector transfected cells (**Figures 3A, C**). Together, EcTRIM23 exerted antiviral action against SGIV and RGNNV *in vitro*.

### EcTRIM23 overexpression enhanced the interferon and inflammatory responses

**Figure 4:**
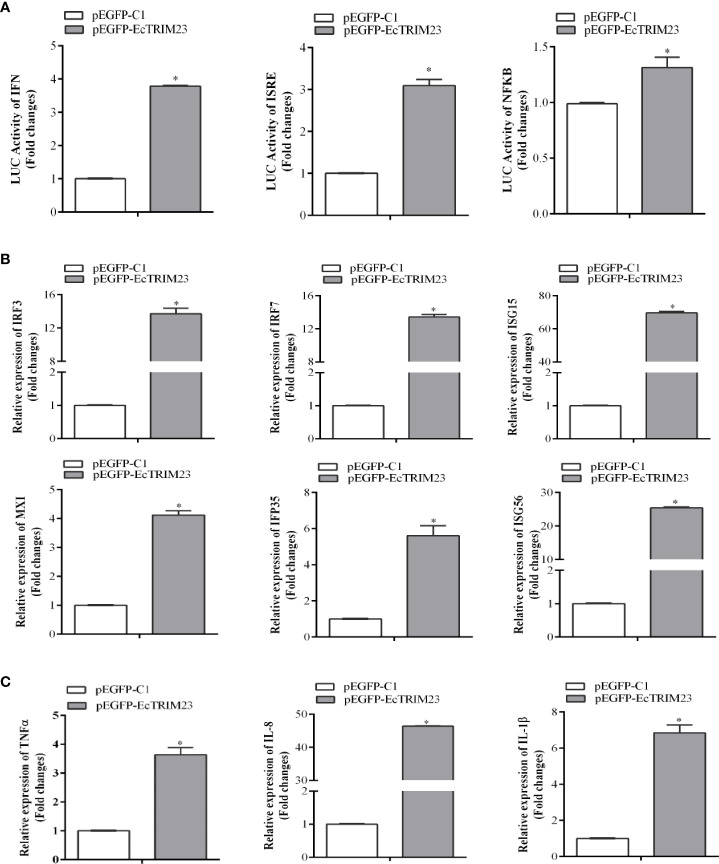
The effects of EcTRIM23 overexpression on host immune response. **(A)** The effects of EcTRIM23 on the promoter activities of IFN, ISRE and NF-κB. GS cells were co-transfected with pEGFP-EcTRIM23 and ISRE-Luc, IFN-Luc, or NF-κB-Luc for 48 h, respectively. Cell were lysed, and the luciferase activities were measured using the dual-luciferase reporter assay. **(B, C)** The effects of EcTRIM23 overexpression on the transcription levels of interferon-related genes **(B)** and pro-inflammatory factors **(C)**. GS cells were transfected with pEGFP-EcTRIM23 or pEGFP-C1, and then the cells were collected at 48 h for qPCR analysis. The expression levels of IRF3, IRF7, ISG15, IFP35, MXI, ISG56, TNF-α, IL-1*β*, and IL-8 were determined by qPCR, respectively. *p < 0.05.

To probe the potential mechanism underlying the antiviral action of EcTRIM23, we firstly detected the effects of EcTRIM23 overexpression on host interferon and inflammatory responses. Using dual-luciferase reporter assay, we found that the promoter activities of ISRE, IFN, and NF-κB in EcTRIM23 transfected cells were all significantly increased compared to those in the cells transfected with the empty vector (**Figure 4A**). Meanwhile, the expression levels of immune-related genes in EcTRIM23-overexpressing cells were examined using qPCR. As shown in **Figure 4B**, the transcripts of interferon related signaling molecules, such as IRF3, IRF7, ISG15, ISG56, IFP35, and MXI were all significantly up-regulated in EcTRIM23-overexpressing cells compared to the control vector transfected cells. In particular, the expression levels of IRF3 and IRF7 in EcTRIM23-overexpressing cells were increased up to 13.87 and 13.53 folds compared to the control vector transfected cells, respectively. In addition, EcTRIM23 overexpression also significantly increased the transcription of pro-inflammatory factors, including TNFα, IL-1β, and IL-8 (**Figure 4C**). The data indicated that EcTRIM23 positively regulated the host interferon and inflammatory responses.

### EcTRIM23 encoded a cytoplasmic protein and interacted with EcTBK1

**Figure 5:**
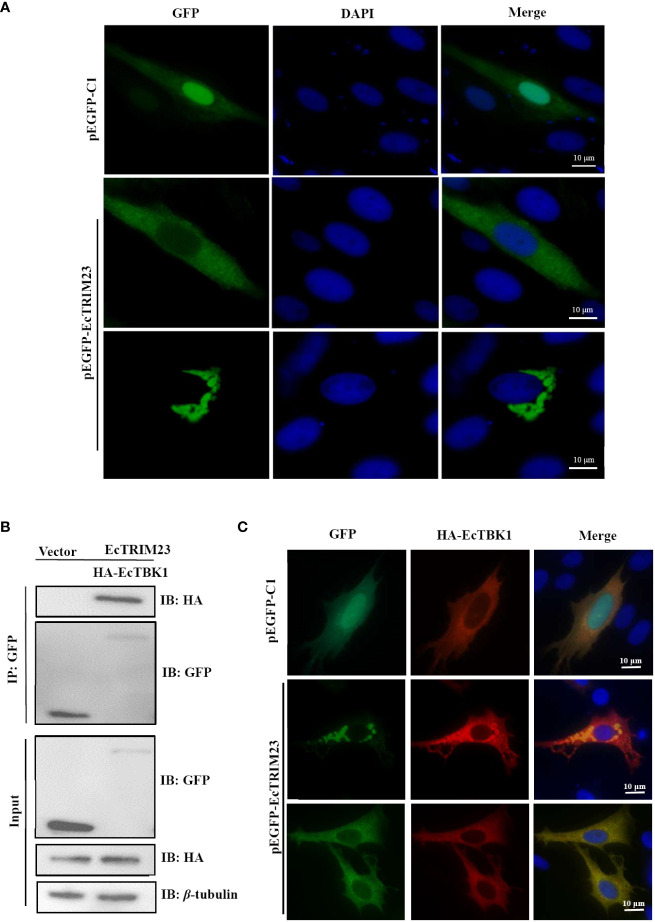
EcTRIM23 recruited and interacted with EcTBK1. **(A)** Subcellular localization of EcTRIM23 in grouper cells. GS cells were transfected with pEGFP-C1 or pEGFP-EcTRIM23 for 48 h After fixation, the cells were stained with DAPI. The fluorescence was observed under fluorescence microscopy. **(B)** EcTRIM23 interacted with EcTBK1. GS cells were co-transfected with pEGFP-EcTRIM23 or pEGFP-C1 and HA-EcTBK1 for 48 h The cells were lysed and immunoprecipitation using the Dynabeads™ Protein G incubation with anti-GFP. Then the Dynabeads-Ab-Ag complex was prepared for immunoblotting assay with anti-GFP and anti-HA antibodies (Abs), respectively. **(C)** EcTRIM23 recruited and colocalized with EcTBK1. GS cells were co-transfected with pEGFP-EcTRIM23 or pEGFP-C1 and HA-EcTBK1 for 48 h, and then fixed for IFA assay.

Next, we detected the subcellular localization of EcTRIM23 *in vitro* under fluorescence microscopy. As shown in **Figure 5A**, the green fluorescence was distributed throughout the cytoplasm and nucleus in the pEGFP-C1 transfected cells. In contrast, the diffuse or aggregated green fluorescence was only observed in the cytoplasm in EcTRIM23-transfected cells, but not in the nucleus. Thus, EcTRIM23 was proposed to encode a cytoplasmic protein.

It has been reported that TRIM23 could interact with TBK1 during its antiviral action (16). Our previous report also showed that EcTBK1 exerted antiviral activity against iridovirus infection (25), thus we probed the potential interaction between EcTRIM23 and EcTBK1 *in vitro.* As shown in **Figure 5B**, Co-IP assay showed that EcTBK1 could only be immunoprecipitated in EcTRIM23-EcTBK1 co-transfected cells, but not in vector-EcTBK1 co-transfected cells, indicating that EcTRIM23 interacted with EcTBK1. Under fluorescence microscope, we observed that the red fluorescence from EcTBK1 evenly distributed in the cytoplasm in pEGFP-C1 and HA-EcTBK1 co-transfected cells, that was consistent with our previous study. Strikingly, EcTRIM23 induced the formation of aggregates of EcTBK1 in some co-transfected cells. Moreover, the diffuse and aggregated fluorescence distribution of EcTRIM23 almost overlapped with that of EcTBK1 (**Figure 5C**), suggesting that EcTRIM23 could recruit and co-localize with EcTBK1.

### EcTRIM23 also interacted with grouper TRAFs

**Figure 6:**
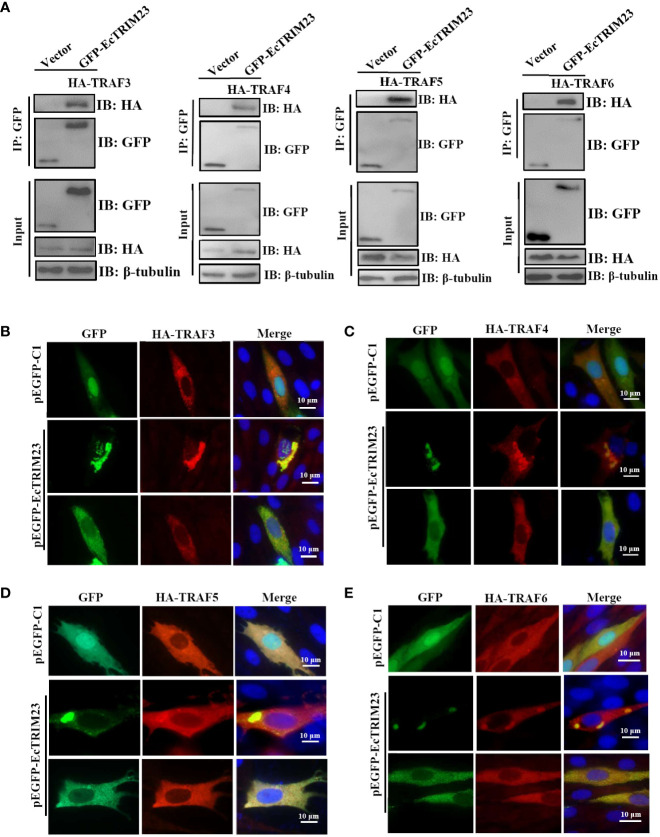
EcTRIM23 interacted with EcTRAFs in grouper cells. **(A)** EcTRIM23 interacted with EcTRAFs, including EcTRAF3, EcTRAF4, EcTRAF5 and EcTRAF6. GS cells were co-transfected with pEGFP-EcTRIM23 or pEGFP-C1 and HA-EcTRAFs for 48 h. After immunoprecipitation with GFP antibody, immunoblotting was carried out using anti-GFP and anti-HA antibodies (Abs), respectively. EcTRIM23 colocalized with different TRAFs, including EcTRAF3 **(B)**, EcTRAF4 **(C)**, EcTRAF5 **(D)** and EcTRAF6 **(E)**. GS cells were co-transfected with pEGFP-EcTRIM23 and HA-EcTRAFs (HA-EcTRAF3, HA-EcTRAF4, HA-EcTRAF5 and HA-EcTRAF6) for 48 h, and then fixed for IFA assay.

TRIM23 has been demonstrated to interact with TRAF6 and TRAF3 during its antiviral action (19, 20). As important cytoplasmic adaptor proteins, TRAFs exert pivotal roles in many biological processes, including immune regulation, inflammatory responses, and apoptosis (39). To ascertain whether EcTRIM23 was associated with EcTRAFs, the interaction between EcTRIM23 and EcTRAFs was also assessed by Co-IP assay and subcellular localization analysis. As shown in **Figure 6A**, in HA-tagged EcTRAFs and pEGFP-EcTRIM23 co-transfected grouper cells, EcTRAF3, EcTRAF4, EcTRAF5 and EcTRAF6 could all be immunoprecipitated with EcTRIM23, but not in vector co-transfected cells. Interestingly, in co-transfected cells, we observed that both the diffuse and aggregated green fluorescence from pEGFP-EcTRIM23 almost overlapped with the red fluorescence recognized HA-tagged EcTRAF3, EcTRAF4, EcTRAF5 and EcTRAF6 (**Figures 6B–E**). These results demonstrated that EcTRIM23 could interact with TRAF3, TRAF4, TRAF5 or TRAF6, respectively.

## Discussion

Increased studies demonstrated that multiple TRIM proteins participated in the distinct steps in the viral life cycle and employed distinct mechanisms to inhibit viral entry, replication or dissemination. Some TRIM proteins modulated signal transduction pathways induced by innate immune sensors, while others were involved in virus-induced autophagy and autophagy-mediated viral clearance (4, 16). Recently, several fish TRIM genes have been shown to be involved in antiviral innate immune response against fish viruses (32–35). Here, the roles of EcTRIM23 in grouper viruses infection were characterized. EcTRIM23 contained the conserved domains from fish to mammals, including RING domain, B-BOX domain, coiled-coil domain, and ARF domain, but showed sequence variations at N-terminal 32 amino acid residues. Upon SGIV or RGNNV infection *in vitro*, the transcription levels of EcTRIM23 were significantly up-regulated, suggested that EcTRIM23 might be involved in SGIV or RGNNV infection. Differently, upon GCRV infection, the expression levels of grass carp TRIM23 were differently regulated in head kidney and spleen (20). Thus, we speculated that fish TRIM23 exhibited different expression profiles against different viruses infection.

Subcellular localization of proteins can provide important information about its physiological function within the cells (40). Members of the TRIM family have different subcellular localization, including filiform, scattered or punctate distributions in the cytoplasm or nucleus (41, 42). In our study, EcTRIM23 showed diffuse and aggregated distribution in cytoplasm which is consistent with the localization of grass crap TRIM23 in CIK cells (20). It has been reported that human TRIM23 was initially associated with the Golgi complex and subsequently localized with lysosome (15). However, we found that EcTRIM23 was not co-localized with ER, Golgi and lysosome (data now shown). Whether the different localizations affects differential functions needed further investigation.

In view of the different roles of TRIM proteins in antiviral immune responses, such as finTRIM82, TRIM62, TRIM35 and TRIM32 (32–35), we firstly evaluated the potential effect of EcTRIM23 on fish virus replication. Our results showed that the overexpression of EcTRIM23 significantly inhibited SGIV and RGNNV replication. Thus, we proposed that EcTRIM23 functioned as an antiviral factor in response to fish RNA virus and DNA infection like EcTRIM8 and EcTRIM25 (27, 33). Further analysis indicated that EcTRIM23 overexpression positively regulated host interferon and inflammatory responses, demonstrated by the up-regulation the transcription of the interferon-related genes and inflammatory-related cytokines. Moreover, the promoter activities of IFN and NFκB were also induced by TRIM23. Our previous studies demonstrated that interferon signaling related molecules, such as IRF3, IRF7 and ISG15 showed antiviral actions against RGNNV or SGIV replication (43–47). Therefore, we speculated that the antiviral function of TRIM23 against grouper viruses might be due to its regulatory roles on these molecules or cytokines. In mammals, TRIM23 was found to act as an antiviral factor by mediating TLR3- and RIG-I/MDA5-mediated antiviral innate and inflammatory responses (17).

Increasing evidences indicate that TRIM23 could exert a potent antiviral state upon virus infection due to its interaction with various proteins, such as TBK1, TRAF3, TRAF6 and MyD88 (18, 20). Here, we evaluated whether that EcTRIM23 interacted with TBK1 and TRAF family members in grouper cells. Our data from Co-IP assay indicated that EcTRIM23 interacted with grouper TBK1, TRAF3, TRAF4, TRAF5 and TRAF6 in grouper cells. Moreover, the ectopic expression of EcTRIM23 induced the aggregates formation of TBK1 and TRAFs in co-transfected cells, and mostly overlapped with them. Our previous studies demonstrated that grouper TBK1 and TRAFs exerted antiviral activity against SGIV or RGNNV infection. Moreover, TBK1 and TRAF3 induced the antiviral and inflammatory responses, evidenced by the significant up-regulation of the antiviral factors, such as IRF3, IRF7 and TNFα (29, 30, 48, 49). Thus, we speculated that the EcTRIM23 might modulate the host interferon and inflammatory responses *via* its interaction with TBK1 or TRAF proteins. In addition, TRIM23 was also found to interact with HSV-1 Us11 protein, and manipulate virus replication (19). Whether EcTRIM23 could regulate fish virus replication through interacting with viral proteins should be clarified in the future study.

In summary, we cloned and investigated the characteristics of EcTRIM23 in the present study. The expression of EcTRIM23 was significantly up-regulated in response to SGIV and RGNNV infection. EcTRIM23 encoded a cytoplasmic protein and served as a crucial antiviral factor against RGNNV and SGIV infection. Furthermore, EcTRIM23 could interact with TBK1, TRAF3, TRAF4, TRAF5 and TRAF6, and finally positively regulated the interferon and inflammatory responses. Our results demonstrated for the first time that fish TRIM23 interacted with multiple TRAF genes, which shed new insights into understanding the function of TRIMs from teleost fish.

## Data availability statement

The datasets presented in this study can be found in online repositories. The names of the repository/repositories and accession number(s) can be found in the article/supplementary material.

## Author contributions

LZ completed the main experiments, analyzed the data, and drafted the manuscript, JZ, WW, SL, and SZ participated in the subcellular localization and qPCR analysis, XH, YH, and QQ designed the projects, conceived and supervised the study, and edited and reviewed the manuscript. All authors contributed to the article and approved the submitted version.

## Funding

This work was supported by grants from the National Key R&D Program of China (2018YFD0900500), National Natural Science Foundation of China (32173007, 31772877), and the earmarked fund for CARS-47-G16.

## Conflict of interest

The authors declare that the research was conducted in the absence of any commercial or financial relationships that could be construed as a potential conflict of interest.

## Publisher’s note

All claims expressed in this article are solely those of the authors and do not necessarily represent those of their affiliated organizations, or those of the publisher, the editors and the reviewers. Any product that may be evaluated in this article, or claim that may be made by its manufacturer, is not guaranteed or endorsed by the publisher.
